# Novel Conopeptides of Largely Unexplored Indo Pacific *Conus* sp.

**DOI:** 10.3390/md14110199

**Published:** 2016-10-27

**Authors:** Eline K. M. Lebbe, Maarten G. K. Ghequire, Steve Peigneur, Bea G. Mille, Prabha Devi, Samuthirapandian Ravichandran, Etienne Waelkens, Lisette D’Souza, René De Mot, Jan Tytgat

**Affiliations:** 1 Toxicology and Pharmacology, KU Leuven, Campus Gasthuisberg, O & N2, Herestraat 49, P.O. Box 922, 3000 Leuven, Belgium; eline.lebbe@pharm.kuleuven.be (E.K.M.L.); steve.peigneur@pharm.kuleuven.be (S.P.); bea.mille@vib-kuleuven.be (B.G.M.); 2 Centre of Microbial and Plant Genetics, KU Leuven, Kasteelpark Arenberg 20, P.O. Box 2460, 3001 Heverlee, Belgium; maarten.ghequire@biw.kuleuven.be (M.G.K.G.); rene.demot@biw.kuleuven.be (R.D.M.); 3 CSIR-National Institute of Oceanography, 403 004 Dona Paula, Goa, India; dprabha@nio.org (P.D.); lisette@nio.org (L.D.); 4 Center of Advanced Study in Marine Biology, Annamalai University, 608 502 Parangipettai, Tamil Nadu, India; sravicas@gmail.com; 5 Laboratory for Protein Phosphorylation and Proteomics, KU Leuven, O & N1 Herestraat 49, P.O. Box 901, 3000 Leuven, Belgium; etienne.waelkens@med.kuleuven.be

**Keywords:** *Conus*, conotoxin, electrophysiology, antimicrobial tests, peptide characterization

## Abstract

Cone snails are predatory creatures using venom as a weapon for prey capture and defense. Since this venom is neurotoxic, the venom gland is considered as an enormous collection of pharmacologically interesting compounds having a broad spectrum of targets. As such, cone snail peptides represent an interesting treasure for drug development. Here, we report five novel peptides isolated from the venom of *Conus longurionis*, *Conus asiaticus* and *Conus australis*. Lo6/7a and Lo6/7b were retrieved from *C. longurionis* and have a cysteine framework VI/VII. Lo6/7b has an exceptional amino acid sequence because no similar conopeptide has been described to date (similarity percentage <50%). A third peptide, Asi3a from *C. asiaticus*, has a typical framework III Cys arrangement, classifying the peptide in the M-superfamily. Asi14a, another peptide of *C. asiaticus*, belongs to framework XIV peptides and has a unique amino acid sequence. Finally, AusB is a novel conopeptide from *C. australis*. The peptide has only one disulfide bond, but is structurally very different as compared to other disulfide-poor peptides. The peptides were screened on nAChRs, Na_V_ and K_V_ channels depending on their cysteine framework and proposed classification. No targets could be attributed to the peptides, pointing to novel functionalities. Moreover, in the quest of identifying novel pharmacological targets, the peptides were tested for antagonistic activity against a broad panel of Gram-negative and Gram-positive bacteria, as well as two yeast strains.

## 1. Introduction

The existence of venomous animals represents a unique starting point for bio-discovery and drug design. Over millions of years, nature has optimized the constituents of venoms (i.e., peptide toxins) as the most selective and potent tools on Earth [1,2]. Therefore, such toxins can be used as lead compounds for a novel generation of drugs. The venom peptides from cone snails (genus *Conus*) are generally small cysteine-rich peptides with the unique feature of being highly selective and potent ligands for a wide range of ion channels and receptors [3]. Consequently, they are recognized as lead compounds in the quest for novel therapeutics in diseases, such as multiple sclerosis, epilepsy, long QT syndrome and many other neurological disorders [4,5].

Conopeptides are classified in two main groups based on the presence and number of cysteine bonds, namely disulfide-rich and disulfide-poor conopeptides. The peptides from the first category are called conotoxins and have multiple disulfide bonds. Peptides from the second category contain none or only one disulfide bond. Disulfide-poor conopeptides are subdivided into contulakines (interacting with neurotensin receptors), conantokines (interacting with *N*-methyl-d-aspartate receptor), conorfamids (interacting with RFamide receptor), conolysines (interacting with cellular membranes), conopressins (interacting with vasopressin receptors), contryphans (interacting with Ca_V_ or K_V_ channels), conophans (target unknown), conomarphines (target unknown) and conomaps (target unknown) [6,7]. The disulfide-rich conotoxins are classified into superfamilies based on a conserved signal sequence and their characteristic cysteine network. Conotoxins with a similar cysteine network carry a similar signal sequence. Further subdivision in families is made based on the receptor with which they interact [6]. These receptors are mainly voltage- and ligand-gated ion channels. Some components act on G-protein coupled receptors and neurotransmitter transporters, whereas others show enzymatic activity [2,7]. To date, at least 16 superfamilies have been discovered [6,7].

Voltage-gated sodium channels (VGSCs or Na_V_s) are transmembrane proteins that are activated by depolarization of the cell membrane. In excitable cells, they play a central role in the generation and propagation of action potentials, in close collaboration with other channels like voltage-gated potassium channels [8]. Nine mammalian channel isoforms of the Na_V_1.X subfamily have been functionally characterized, and several insect channels have been successfully cloned and expressed [9]. The mammalian Na_V_ isoforms have similar structural and functional properties, but are present in different cell types (neurons, neuro-endocrine cells, skeletal muscle cells, heart cells); and they possess diverse functional properties in the corresponding tissues [10]. Furthermore, defective Na_V_s cause several diseases or channelopathies, such as epileptic disorders, neuromuscular diseases, cancer and cardiomyopathies. Blocking the aberrant Na^+^ current can be an effective strategy in treating these disorders [11].

The molecular diversity of K^+^ channels is larger than any other group of ion channels, with more than 80 different genes and many splice variants [12]. Voltage-gated potassium channels (VGPC or K_V_s) are responsible for the repolarization of membranes following a neuron-initiated action potential. K_V_ channels are specifically and widely distributed and are located in the brain, nervous system, heart, skeletal muscle, hematopoietic cells, lymphocytes and osteoclasts [13]. They are involved in many physiological processes, such as regulation of heart rate, neuronal excitability, muscle contraction, neurotransmitter release, insulin secretion, Ca^2+^ signaling, cellular proliferation and migration and cell volume regulation [14].

Nicotinic acetylcholine receptors (nAChRs) are a member of the ligand-gated cationic channel family and mediate fast synaptic transmission. They are broadly distributed throughout the peripheral and central nervous systems of both simple and evolutionarily complex organisms [15]. In mammals, there are 16 different nAChR subunits: nine different α-subunits (α_1–7_, α_9_ and α_10_), four β-subunits (β_1–4_), as well as γ, δ and ε subunits. Five of these subunits combine to form muscle nAChR subtypes (α_1_β_1_γδ and α_1_β_1_δε), which are found at neuromuscular junctions, whereas the rest (α_2_–α_10_, β_2_–β_4_) assembles in numerous homomeric (α-subunits exclusively) or heteromeric (α- and β-subunits) neuronal nAChR subtypes [16]. The assembly of different pentamers forms a complex variety of nAChR subtypes with different pharmacological and biophysical properties [17].

This study describes the isolation and purification of novel conopeptides from *Conus longurionis*, *Conus asiaticus* and *Conus australis* that were collected from the Tamil Nadu coast, in the Indian Ocean. To the best of our knowledge, this is the first report of a conopeptide from the venom of *C. asiaticus*, while other conotoxins from *C. longurionis* and *C. australis* have been described earlier [18,19]. Toxins were electrophysiologically screened against a panel of Na_V_s, K_V_s, as well as nAChRs. Moreover, in the quest of identifying novel pharmacological targets of conopeptides, we tested these peptides for potential antimicrobial activity.

## 2. Results

### 2.1. Isolation of Novel Conotoxins from C. longurionis, C. asiaticus and C. australis

Venom glands of three largely unexplored cone snail species, *C. longurionis*, *C. asiaticus* and *C. australis*, were investigated. Samples were purified via a series of HPLC purification steps. Amino acid sequences of the purified compounds were determined via *N*-terminal Edman degradation, revealing five new conotoxin sequences (Table 1).

The first peptide, Lo6/7a is a novel 24-residue conotoxin with a molecular mass of 2583.0 Da (folded). This is in perfect agreement with the mass of the unfolded synthetic peptide (2589.0 Da), determined by LC-MS and the theoretically calculated masses for oxidized (2583.0 Da) and reduced (2589.0 Da) Lo6/7a. According to its cysteine pattern, C–C–CC–C–C, Lo6/7a belongs to framework VI/VII, covered by different superfamilies: I, O and M.

**Figure 1:**
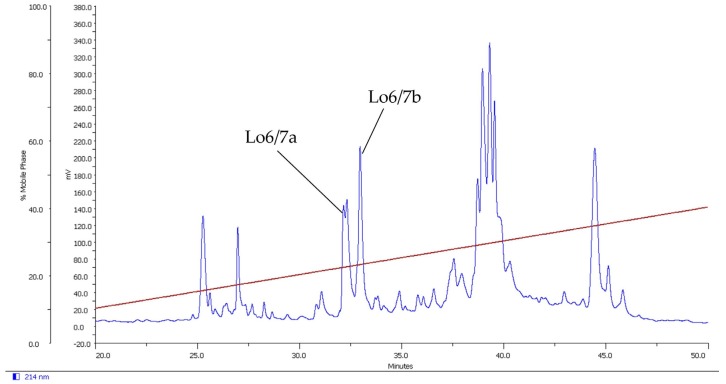
C18 RP-HPLC purification of *C. longurionis*, showing peptides Lo6/7a and Lo6/b. The brown line shows the acetonitrile gradient.

The second peptide, Lo6/7b, has a molecular mass of 2775.1 Da (folded peptide) determined by MALDI-TOF. This mass resembles the mass of the unfolded synthetic peptide (2781.1 Da), determined by LC-MS and the theoretically calculated masses of oxidized (2775.1 Da) and reduced (2781.1 Da) Lo6/7b. The cysteine pattern of Lo6/7b is the same as for Lo6/7a, C–C–CC–C–C and therefore also belongs to superfamilies I, O and M and framework VI/VII. The RP-HPLC purification chromatogram of *C. longurionis* venom is shown in Figure 1.

Asi3a and Asi14a were retrieved from *C. asiaticus*, a worm hunting cone snail species found in the Indian Ocean at the coast of Tamil Nadu (India) and represent the first conopeptides isolated from this species. The molecular mass of Asi3a is 1697.6 Da (folded) obtained by MALDI-TOF which is in perfect agreement with the mass of the synthetic unfolded peptide 1703.0 Da and the theoretical masses of folded Asi3a (1696.1 Da) and unfolded (1702.1 Da) peptide. Asi3a has cysteine framework CC–C–C–CC, characteristic for framework III, found in the M-superfamily.

**Figure 2:**
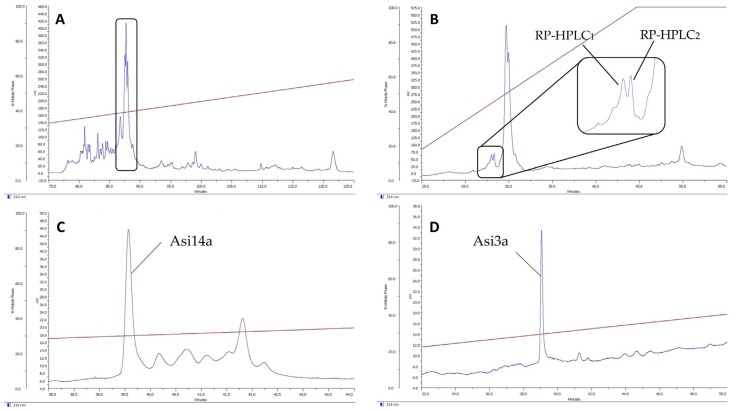
Purification of crude venom of *C. asiaticus*. (**A**) RP-HPLC chromatogram of *C. asiaticus* venom. The peak indicated in the box was collected for further purification; (**B**) Ion exchange chromatogram of the peak fractions collected in the first purification step. The indicated peaks were subjected to another RP-HPLC purification step; (**C**) RP-HPLC_1_ chromatogram as indicated in (**B**). Edman degradation of the first peak, Asi14a, revealed the amino acid sequence as described; (**D**) RP-HPLC_2_ chromatogram, as indicated in Figure 2B. Edman degradation of this peak, Asi3a, revealed the amino acid sequence as described above. The brown lines in (**A**,**C**,**D**) are acetonitrile gradients. The line in (**B**) shows the ion exchange gradient.

Asi14a has a molecular mass of 1697.6 Da (folded), determined by MALDI-TOF. The mass of the unfolded synthetic peptide is 1700.0 Da. The calculated mass of oxidized Asi14a is 1695.9 Da, and the reduced calculated mass is 1699.9 Da. Asi14a has a framework XIV cysteine arrangement (C–C–C–C), as found in the A, L and J superfamilies. The purification of the crude venom of *C. asiaticus* is shown in Figure 2. The RP-HPLC chromatogram of the first purification step is shown in Figure 2A. In Figure 2B, the ion exchange chromatogram is shown, whereas a third purification via RP-HPLC revealed the purified peptides Asi14a (Figure 2C) and Asi3a (Figure 2D).

**Figure 3:**
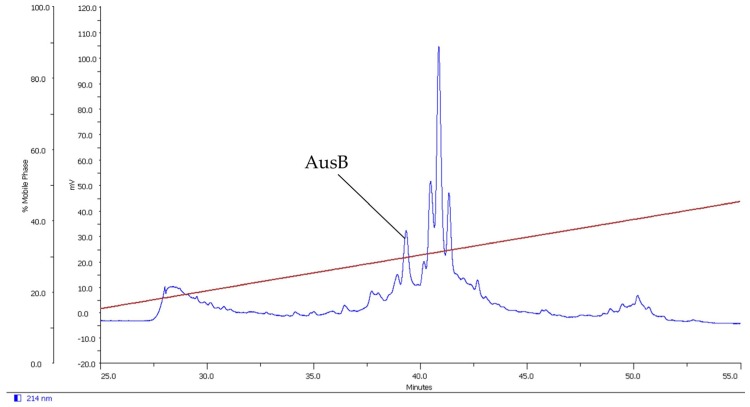
RP-HPLC purification of AusB (gel filtration fraction). The brown line shows the acetonitrile gradient.

The last peptide was found in the venom of *C. australis* and was named AusB. It has a molecular mass of 2030.8 Da, determined by MALDI-TOF. The molecular mass of the unfolded synthetic peptide is 2032.2 Da, determined by LC-MS, and correlates with the calculated masses of the oxidized peptide (2030.2 Da) and for reduced AusB (2032.2 Da). Figure 3 shows the RP-HPLC purification chromatogram.

### 2.2. Electrophysiological Screening against Voltage-Gated and Ligand-Gated Ion Channels

The masses of the synthetic peptides were determined by MALDI-TOF, validating that the peptides were folded successfully. Peptides were purified and electrophysiologically screened against a panel of Na_V_, K_V_ and Ca_V_ channels, as well as nAChRs, according to their framework.

#### 2.2.1. Lo6/7a and Lo6/7b

**Figure 4:**
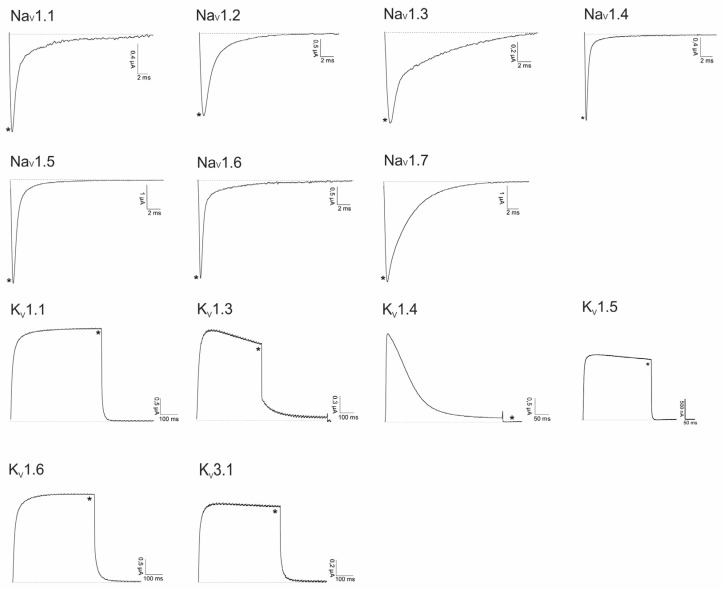
Electrophysiological screening of Lo6/7a and Lo6/7b. * Represents traces after toxin application (1 μM) and overlapping control traces before toxin application.

Lo6/7a and Lo6/7b have a cysteine framework VI/VII, and both belong to the O or I3- superfamily according to the conotoxin classification described by Akondi et al. [2]. Therefore, the folded peptides were screened against a panel of Na_V_ and K_V_ channels (Figure 4). Preliminary screening on Ca_V_ channels did not reveal significant inhibition of the channel (results not shown). Therefore, up to now, no target could be assigned to Lo6/7a and Lo6/7b.

#### 2.2.2. Asi3a

**Figure 5:**
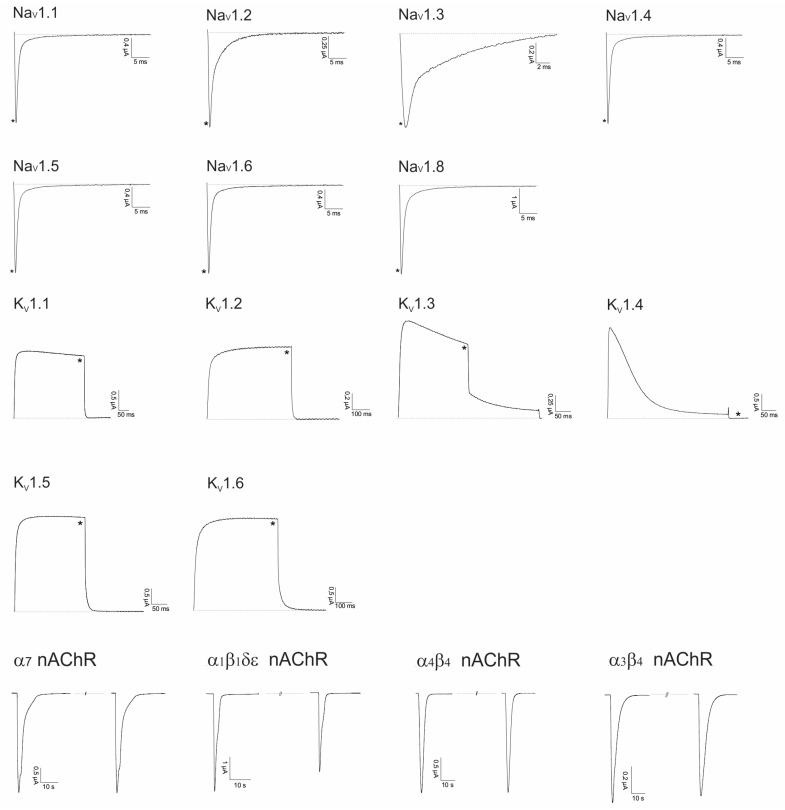
Electrophysiological screening of Asi3a on Na_V_ and K_V_ channels (10 μM) and nAChRs (5 μM). * Represents traces after toxin application and overlapping control traces before toxin application.

Asi3a belongs to the M-superfamily from which other members target Na_V_s, K_V_s or nAChRs. Therefore, we performed an electrophysiological screening against a panel of these channels, visualized in Figure 5. None of the tested ion channels was influenced by this peptide.

#### 2.2.3. Asi14a

**Figure 6:**
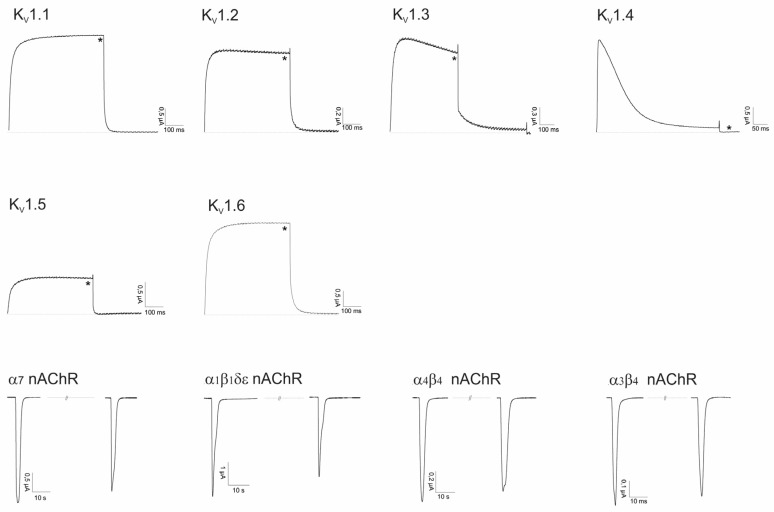
Electrophysiological screening of Asi14a on K_V_ channels (10 μM) and nAChRs (5 μM). * Represents traces after toxin application and overlapping control traces before toxin application.

Asi14a is another peptide found in the venom from *C. asiaticus*. Conopeptides from the J- or L-superfamily mainly act on nAChRs and K_V_s. Therefore, we electrophysiologically screened Asi14a against a selected panel of these channels (Figure 6). No antagonistic activity of Asi14a could be observed.

#### 2.2.4. AusB

**Figure 7:**
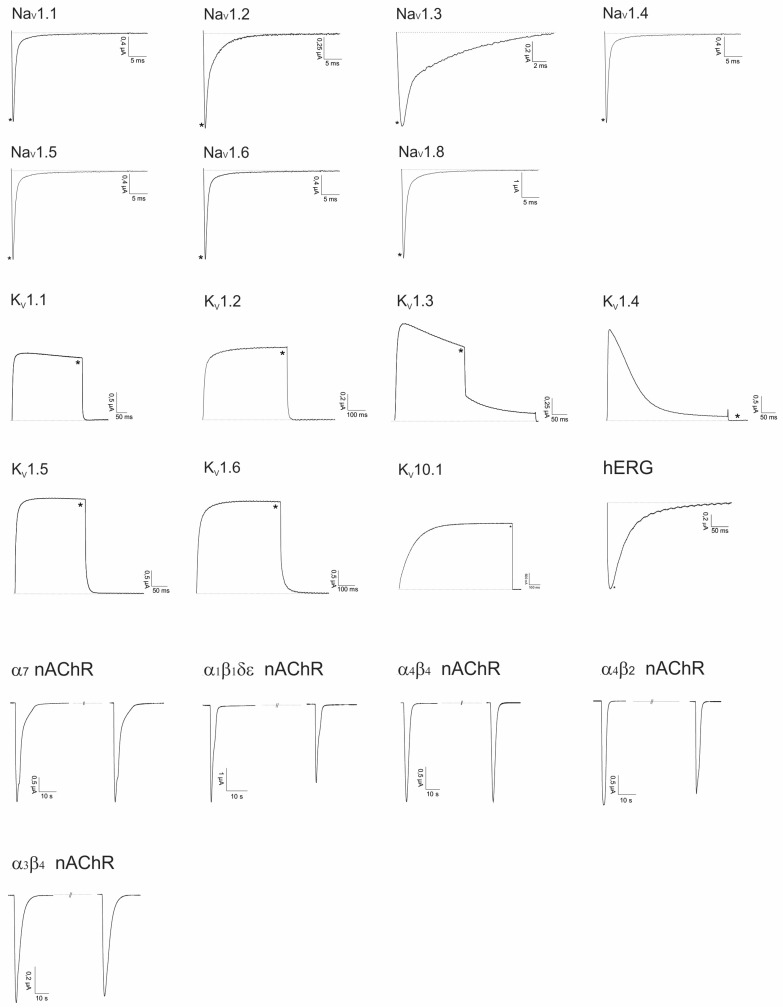
Electrophysiological screening of AusB (10 μM) on a panel of Na_V_, K_V_ and nAChRs. * Represents traces after toxin application and overlapping control traces before toxin application.

AusB from *C. australis* is a disulfide poor peptide, having only one Cys-bond. Since no function was previously assigned to such conopeptides, its effect on a large panel of channels was evaluated. The peptide was electrophysiologically screened against a panel of Na_V_s, K_V_s and nAChRs as expressed heterologously in *Xenopus laevis* oocytes (Figure 7). Up to now, no target could be identified.

### 2.3. Antibacterial Activity

The five new conopeptides were screened against 29 Gram-negative and 10 Gram-positive bacterial strains and two yeast strains (Table 2). A turbid zone of inhibition was only obtained for Lo6/7a against the Gram-positive strain *Bacillus megaterium* ATCC13632. For the other peptides, no growth inhibition was observed.

## 3. Discussion

In this report, we describe five novel conopeptides, discovered in the venom of *C. longurionis*, *C. asiaticus* and *C. australis*. These novel sequences have different cysteine frameworks and some of them likely represent new subgroups, based on sequence comparison with known conotoxins.

### 3.1. Conopeptide Alignment and Classification

**Figure 8:**
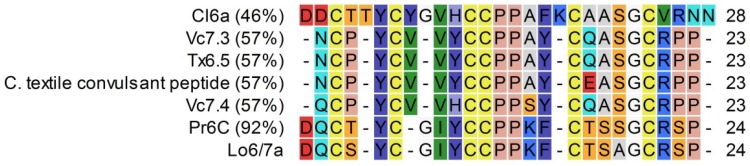
Alignment of Lo6/7a with conotoxins, sorted by the percentage of identity (indicated in the left column). References: Pr6c (*C. parius*, [22]), Vc7.4 (*C. victoriae*, [21]), a convulsant peptide from *C. textile* [20,23], Tx6.5 (*C. textile*, [24]), Vc7.3 (*C. victoriae*, [21]) and Cl6a (*C. californicus*, [25]).

Peptide Lo6/7a is a 24-residue conotoxin isolated from the venom of *C. longurionis*. Depending on the target, peptides from the O-superfamily are subdivided into different families: ω-conotoxins act on Ca_V_ channels; κ-conotoxins target K_V_ channels; and μO- or δ-conotoxins influence Na_V_ channels. By performing a Conoserver alignment search on Lo6/7a, the highest percentage of similarity (92%) was obtained for Pr6c from *Conus parius* [8] (Figure 8). Up to now, the target from Pr6c also remains to be discovered, but the authors suggested the peptide to be either an ω- or κ-conotoxin. Despite the high sequence identity of Lo6/7a with Pr6c, we could not demonstrate such activity on Ca_V_ or K_V_ channels unequivocally. Another peptide from *Conus textile* (a peptide causing convulsions in mice) has a similarity percentage of 59% [20]. This peptide induces symptoms characterized by ‘‘sudden jumping activity followed by convulsions, stretching of limbs and jerking behavior’’. The authors predicted that this peptide belongs to a new undefined class of conotoxins. Two other peptides, Vc7.4 and Vc7.3, from *Conus victoriae* were described by Robinson et al. (2014) [21]. In this study, the precursor sequences of Vc7.4 and Vc7.3 were identified, and it was shown that these peptides, as well as the textile convulsant peptide (*C. textile*), are members of a previously-undefined conotoxin superfamily, which was designated U-superfamily. This peptide superfamily shares the cysteine framework (VI/VII) of most members of the O1-, O2- and O3-superfamilies. However, the pre- and pro-peptide sequences substantially differ from other known conotoxin superfamilies. Moreover, when the O-superfamily is compared with the U-superfamily, there is little similarity in the intercysteine loop composition or length (i.e., the U-superfamily has only two residues, while the O-superfamily conotoxins have six) [21]. The specific physiological target of these peptides has not yet been derived. However, given the similarity in the mature peptide sequence of these conotoxins with Lo6/7a, it is likely that they belong to the same superfamily and share a similar target.

**Figure 9:**
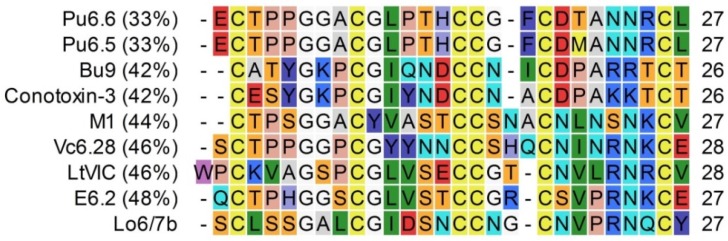
Alignment of Lo6/7b with conotoxins, sorted by percentage of identify (indicated in the left column). References: E6.2 (*C. ermineus*, unknown ref.); LtVIC (*C. litteratus*, [26]); Vc6.28 (*C. victoriae*, [21]); M1 (*C. militaris*, [27]); Conotoxin-3 (*C. striatus*, [28]); Bu9 (*C. bullatus*, [29]); Pu6.5 (*C. pulicarius*, [30]); Pu6.6 (*C. pulicarius*, [30]).

Peptide Lo6/7b aligns with members of the O-superfamily, although with low percentages of similarity (Figure 9). LtVIC is the only peptide from which a physiological target has been identified up to now. This conotoxin inhibits sodium currents in adult rat dorsal root ganglion neurons [26]. Therefore, LtVIC is considered a μ(*O*)-conotoxin. In our electrophysiological set-up, we could not identify Lo6/7b as a μ(*O*)-conotoxin. Nevertheless, the similarity of Lo6/7a with LtVIC (46%) is rather low.

**Figure 10:**
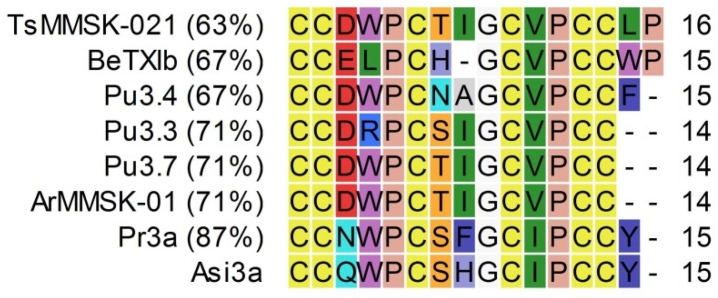
Alignment of Asi3a with conotoxins, sorted by percentage of identity (indicated in the left column). References: Pr3a (*C. parius*, [22]), ArMMSK-01 (*C. arenatus*, [31]); Pu3.7 (*C. pulicarius*, [30]); Pu3.3 (*C. pulicarius*, [24]); Pu3.4 (*C. pulicarius*, [32]); BetTXIb (*C. betulinus*, [33]); TsMMSK-021 (*C. tessulatus*, [31]).

Asi3a is classified in the M-superfamily, acting generally on Na_V_s (μ-conotoxins), K_V_s (κM-conotoxins) and nAChRs (ψ-conotoxins). Asi3a shows most identity with conotoxin Pr3a from *Conus parius* (87%) (Figure 10). Jimenez et al. [22] classified this peptide as an M-superfamily conotoxin and performed a bioassay that was carried out by intraperitoneal injection of fish. The purified peptide Pr3a (1 nmol) resulted in paralysis of the fish after ~5 min. A functional characterization of peptides similar to Asi3a has not yet been performed.

Asi14a belongs to the A-, L- or J- superfamilies acting typically on nAChRs (L- and A-superfamily). The J-superfamily characteristically targets K_V_ channels. No meaningful alignment with other A-, L- or J-superfamily peptides could be performed. Therefore, we conclude that Asi14a probably belongs to a new subclass of framework XIV proteins. A BLAST homology search with Asi14a did not reveal similarity to any known peptide or protein.

AusB is an unusual peptide found in the venom of *C. australis*. Containing 18 amino acids, AusB has only one cysteine bond classifying it among the disulfide poor conopeptides. A Conoserver search resulted in a poor quality alignment, and a BLAST did not align the peptide with other relevant peptides either. Since AusB could not be matched with any disulfide-poor conotoxin, this peptide represents a new family, which we will label ConoGAY peptides, named after the first three *N*-terminal amino acids of this peptide.

### 3.2. Antagonistic Assays in the Quest of Identifying Novel Pharmacological Targets

Literature indications for conotoxins as potential antimicrobial compounds are given by Biggs et al. [34], Jiang et al. [35] and Takada et al. [36]. Biggs et al. (2007) discovered conolysin-Mt, a disulfide-poor conopeptide that was initially tested on oocytes where it causes membrane potential collapse within seconds. The peptide was also evaluated for antagonism against three bacterial strains: *E. coli* D21, *E. coli* ATCC 25922 and *S. aureus* ATCC 6538. The authors noticed low antibacterial activity against the two *E. coli* strains tested, with a minimal inhibitory concentration (MIC) > 50 μM. The MIC against the Gram-positive *S. aureus* was 25–50 μM [34]. Jiang et al. (2011) tested a cysteine-rich peptide library mimicking μ-conotoxins from *Conus geographus* on antiviral activity against influenza virus [35]. Finally, Takada et al. (2006) showed that asteropine A, a sialidase-inhibiting conotoxin-like peptide from the marine sponge *Asteropus simplex*, might be an important lead compound for antibacterial and antiviral drug development [36]. This is interesting since multidrug resistant bacterial infections are a growing global health problem. Antimicrobial peptides from poisonous animals are described for a number of scorpion peptides, as well as peptides from snakes, frogs, bees (*Apis* sp.), etc., as part of their host defense system [37,38,39,40,41,42,43,44,45,46]. For scorpions in particular, it has been proposed that the presence of antibacterial peptides protects the venom gland from pathogenic infections or potentiates toxin action [47]. Scorpion antimicrobial peptides (AMP) are positively-charged amphipathic peptides divided into three structural categories: (1) cysteine containing peptides with mainly three or four disulfide bridges; (2) peptides with an amphipathic α-helix, but lacking cysteine residues; and (3) peptides rich in Pro and Gly amino acids. One example of a cysteine containing scorpion AMP is scorpine, which showed activity against both Gram-positive (*B. subtilis*) and Gram-negative (*K. pneumonia*) bacteria (MIC 1–10 μM) [47]. When it comes to conotoxins, this path of investigation remains underexplored.

In this work, all peptides were electrophysiologically tested on relevant ion channels predicted by their cysteine arrangement. These results indicate novel functionalities other than expected based on their cysteine framework, since no activity could be identified on all of the targets studied. In order to further determine the mode of action and the potential molecular targets of these conotoxins, in vivo or ex vivo assays should be performed. As such, symptoms observed after intracranial injection of toxins in mice may provide indications on the type of receptor or channel targeted. Furthermore experiments on neuromuscular preparations may possibly identify a pre- or post-synaptic effect or even sodium/potassium or nicotinic antagonism. In addition, a broad screening was performed against a collection of micro-organisms. Low and very specific activity was observed for Lo6/7a against *Bacillus megaterium* ATCC13632. Since 1 mM is a very high test concentration and the halo was small, the inhibitory effect of Lo6/7a cannot be attributed as the main action of this peptide. Examples of scorpion antimicrobial peptides that potently target *B. megaterium* are meucin-13 (MIC 0.25 μM), meucin-18 (MIC 0.25 μM) and pantinin-3 (MIC 6 μM) [47].

## 4. Materials and Methods

### 4.1. Cone Snail Specimens and Venom Extraction

Specimens of *C. longurionis*, *C. asiaticus* and *C. australis* (identified by Kiener (1845), da Motta (1985) and Holten (1802), respectively, and classified by Tucker and Tenorio [48]) were collected from the Indian Ocean near Tamil Nadu, India. The venomous apparatuses (venom bulbs and venom ducts) were extracted from the specimens as described previously [49]. The collected tissues were preserved in RNAlater solution (Ambion, Austin, TX, USA) and stored at −20 °C. The venomous apparatuses were used for peptide/protein extraction.

### 4.2. Peptide Fractionation and Purification

Two steps were followed for the separation of the venom compounds of *C. longurionis*. In the first step, the lyophilized crude venom powder was solubilized into 50% acetonitrile (ACN)/water and aliquots were loaded on a Gel filtration Superdex™ Peptide 10/300 GL column with 50% ACN/water as the mobile phase (flow rate 0.5 mL/min) to separate the peptides and proteins based on their size. Two sample collections obtained were stored overnight at −80 °C, freeze-dried and finally solubilized in 5% ACN/water. For the second step, an analytical Vydac C18 column (218MS54, 4.6 mm × 250 mm, 5-μm particle size; Grace, Deerfield, IL, USA) with a two solvent system was used: (A) 0.1% trifluoroacetic acid (TFA)/H_2_O and (B) 0.085% TFA/ACN. The sample was eluted at a constant flow rate of 1 mL/min with a 0%–80% gradient of Solvent B over 90 min (1% can per minute after 10 min of Solvent A). The HPLC column elutes were monitored by a UV/VIS-155 detector (214 nm and 280 nm; Gilson, Middleton, WI, USA).

Three steps were followed for the separation of the venom compounds of *C. asiaticus*. In the first step, the lyophilized crude venom powder was solubilized using 5% acetonitrile (ACN)/water and aliquots were loaded on an analytical Vydac C18 column (218MS54, 4.6 mm × 250 mm, 5-μm particle size; Grace, Deerfield, IL, USA) with a two-solvent system: (A) 0.1% trifluoroacetic acid (TFA)/H_2_O and (B) 0.085% TFA/ACN. The sample was eluted at a constant flow rate of 1 mL/min with a 0%–80% gradient of Solvent B over 90 min (1% ACN per minute after 10 min of Solvent A). The HPLC column elutes were monitored by a UV/VIS-155 detector (214 nm and 280 nm; Gilson, Middleton, WI, USA). The largest peak was collected and freeze-dried for further purification. This fraction collection was subjected to a second purification step, namely ion exchange chromatography, using a Luna SCX column (Phenomenex, Torrance, CA, USA, 4.6 mm × 250 mm, 5-μm particle size) at room temperature. The solutions used for ion exchange chromatography were: (A) 20 mM KH_2_PO_4_, pH 2.5: ACN (75:25) and (B) 20 mM KH_2_PO_4_/0.5 M KCl:ACN (75:25). The sample was eluted using a three-step protocol: 0% Solution B for 15 min, 0%–100% Solution B for 30 min and 100% B for 15 min at a flow rate of 1 mL/min. The collected fractions were stored overnight at −80 °C and freeze-dried. A third purification step was performed by RP-HPLC, using the same conditions as described for the first purification step.

Two steps were followed for the separation of the venom compounds of *C. australis*. In the first step, the lyophilized crude venom powder was solubilized into 50% acetonitrile (ACN)/water, and aliquots were loaded on a Gel filtration Superdex™ Peptide 10/300 GL column with 50% ACN/water as the mobile phase (flow rate 0.5 mL/min) to separate the peptides and proteins based on their size. Three sample collections were made that were stored overnight at −80 °C, freeze-dried and finally solubilized in 5% ACN/water. For the second step, an analytical Vydac C18 column (218MS54, 4.6 mm × 250 mm, 5-μm particle size; Grace, Deerfield, IL, USA) with a two-solvent system was used: (A) 0.1% trifluoroacetic acid (TFA)/H_2_O and (B) 0.085% TFA/ACN. The sample was eluted at a constant flow rate of 1 mL/min with a 0%–80% gradient of Solvent B over 90 min (1% ACN per minute after 10 min of Solvent A). The HPLC column elutes were monitored by a UV/VIS-155 detector (Gilson, Middleton, WI, USA) scanning both 214 nm and 280 nm.

### 4.3. Peptide Sequencing

Isolated Lo6/7a, Lo6/7b, Asi3a, Asi14a and AusB were collected and freeze-dried for direct peptide sequencing and molecular mass analysis (MALDI-TOF; 4800 Analyzer, Applied Biosystems, Foster City, CA, USA). A Protein Sequencer PPSQ-31A/33A (Shimadzu, Kyoto, Japan) was used to determine the amino acid sequence of the separated compounds. Samples were loaded onto a polybrene-pretreated, precycled glass fiber disk and Edman sequenced for 30 residue cycles.

Theoretically-calculated masses of the peptides were done with an online Peptide Mass Calculator (Peptide Protein Research Ltd., Hampshire, UK). Peptide homology search was generated online at Conoserver.org [50,51] and NCBI (Rockville Pike, Bethesda MD, USA) [52]. The CLC Main Workbench 7 software was used to align the peptide sequences (CLC bio, QIAGEN, Hilden, Germany).

### 4.4. Peptide Synthesis and Folding

Lo6/7a and Lo6/7b were synthesized by GeneCust (Elange, Luxemburg). Asi3a, Asi14a and AusB were synthesized by GenicBio Limited (Shanghai, China). All peptides, except AusB, were *C*-terminally amidated, purified by HPLC and analyzed with LC-MS, then freeze-dried and stored at −20 °C until use. The peptides were folded using an oxidative folding solution (1 mM reduced glutathione (Sigma, Munich, Germany), 1 mM oxidized glutathione (Roche, Mannheim, Germany), 1 mM ethylenediaminetetraacetic acid (EDTA; Sigma, Munich, Germany) and 100 mM Tris/HCl (Merck, Darmstadt, Germany) [53]). The solution was adjusted to pH 7.63 with 10 M NaOH (Merck, Darmstadt, Germany). Prior to functional characterization, the purity and folding of the synthetic peptides was validated (MALDI-TOF MS), and a chromatographic characterization was undertaken by RP-HPLC. On the basis thereof, a careful comparison of the retention time with the one of the native material was done.

### 4.5. Preparation of RNA for Functional Testing in Xenopus Oocytes

The RNA preparation of the different Na_V_, K_V_ channels and nAChRs was done as follows:

Complementary DNA (cDNA) encoding the Na_V_ channels was subcloned into the corresponding vector: the α-subunits rNa_V_1.1/pLCT1 (NotI), rNa_V_1.2/pLCT1 (NotI), rNa_V_1.3/pNa3T (NotI), rNa_V_1.4/pUI-2 (NotI), hNa_V_1.5/pcDNA3.1 (XbaI), mNa_V_1.6/pLCT (NotI), rNa_V_1.7/pBSTA.rPN1 (SacII), hNa_V_1.8/hPN3-pBSTAcIIR (NotI) and the corresponding β-subunits rβ_1_/pSP64T (EcoRI) and hβ_1_/pGEM-HE (NheI).

For K_V_ channels, channel-encoding cDNA was subcloned into the corresponding vector: rK_V_1.1/pGEM-HE (EcoRI), rK_V_1.2/pGEM-HE (SphI), hK_V_1.3/pGEM-HE (NotI), rK_V_1.4/pGEM-HE (NotI), rK_V_1.5/pGEM-HE (SalI), rK_V_1.6/pGEM-HE (NdeI), hK_V_3.1/pGEM-HE (XbaI), hK_V_10.1/pSGem (SfiI), hERG/pSp64 (EcoRI).

Complementary DNA encoding the nAChR-channels was subcloned into the corresponding vector: hα_3_/pcDNA3 (XbaI), hα_4_/pGEM-HE (NheI), cα_7_/pBlueScript (NotI), hβ_2_/pSP64 (PvuII), hβ_4_/pcDNA3 (XbaI), rα_1_/pSP0oD (SalI), rβ1/pSP0oD (SalI), rδ/pSP0oD (SalI), rε/pSP0oD (SalI).

### 4.6. Electrophysiological Recordings

The harvesting of Stages V–VI oocytes from anaesthetized female *Xenopus laevis* frogs was described previously [54]. Using a nanoinjector (Drummond, Broomall, PA, USA), the selected oocytes (Stages V–VI) were injected with 40–70 nL mRNA (100–2000 ng/L) and 4–20 nL mRNA for K_V_ channels. Then, the oocytes were stored at 16 °C in a geomycin (1.25 mL/L; Rotexmedica, Trittau, Germany) and theophylline (80 mg/L; ABC chemicals, Wauthier Braine, Belgium) supplemented ND96 solution, except for K_V_ channels, where theophylline was not added.

Whole-cell currents from oocytes were recorded at room temperature (18–22 °C) by the two-electrode voltage clamp technique using a GeneClamp 500 amplifier (Axon Instruments, Foster City, CA, USA) controlled by a pClamp data acquisition system (Molecular Devices, Sunnyvale, CA, USA). Oocytes were placed in a bath containing ND96 solution. Voltage and current electrodes were filled with 3 M KCl, and the resistances of both electrodes were maintained as low as possible (0.5 to 1.5 MΩ). To eliminate the effect of the voltage drop across the bath grounding electrode, the bath potential was actively controlled by a two-electrode bath clamp. Leak subtraction was performed using a −P/4 protocol.

For Na_V_ channels, whole-cell current traces were evoked every 5 s by a 100-ms depolarization to the voltage corresponding to the maximal activation of the Na_V_-subtype in control conditions (0 mV), starting from a holding potential of −90 mV. The elicited currents were sampled at 20 kHz and filtered at 2 kHz using a four-pole, low-pass Bessel filter. Concentration-response curves were constructed by adding different toxin concentrations directly to the bath solution.

K_V_1.1–K_V_1.6 and K_V_3.1 currents were evoked by 500-ms depolarizations to 0 mV followed by a 500-ms pulse to −50 mV, from a holding potential of −90 mV. The elicited currents were sampled at 2 kHz and filtered at 500 Hz using a four-pole low-pass Bessel filter. K_V_10.1 currents were evoked by 2-s depolarizing pulses to 0 mV from a holding potential of −90 mV. hERG or K_V_11.1 peak and tail currents were generated by a 2.5-s prepulse from −90 mV–40 mV followed by a 2.5-s pulse to −120 mV. K_V_10.1 currents were sampled at 2 kHz and filtered at 1 kHz; hERG currents were sampled at 10 kHz and filtered at 1 kHz using a four-pole low-pass Bessel filter.

For measuring nAChR currents, the following conditions were applied: during recordings, oocytes were continuously perfused with ND96 at a rate of 2 mL/min, with the conopeptides applied during 30 s before ACh was added. ACh (200 μM) was applied for 2 s at 2 mL/min, with 30-s washout periods between different ACh applications and 200 s after toxin application. The percentage response or percentage inhibition was obtained by averaging the peak amplitude of at least three control responses (two directly before exposure to the peptide and one after 200 s washout). Whole-cell current traces were evoked from a holding potential of −90 mV.

Data were analyzed using pClamp Clampfit 10.0 (Molecular Devices, Sunnyvale, CA, USA) and Origin 7.5 software (Originlab, Northampton, MA, USA) and presented as the result of at least 3 independent experiments (*n* ≥ 3).

### 4.7. Growth Media and Micro-Organisms

Growth media were prepared as follows (1 L deionized water):

LB (lysogeny broth): 0.5% yeast extract (BD Biosciences, San Jose, CA, USA); 1% bacterial peptone (International Medical); 1% NaCl (Fisher Scientific, Aalst, Belgium); MRS (Man Rogosa and Sharpe): 55 g Difco™ lactobacilli MRS broth (BD Biosciences, San Jose, CA, USA); NB (nutrient broth): 8 g nutrient broth (Oxoid); TSB (trypticase soy broth): 3%, BD Biosciences; TY (tryptone yeast extract): 3 g yeast extract, 5 g tryptone (International Medical); 7 mM CaCl_2_ (VWR International, Leuven, Belgium); YEP (yeast extract peptone): 10 g yeast extract, 10 g bacterial Bacto peptone; YPD (yeast extract peptone dextrose): 10 g yeast extract, 20 g bacterial peptone, 20 g dextrose. Media were solidified with agar (1.5%) For this study, 29 Gram-negative and 10 Gram-positive strains were used, together with 2 yeast strains. Strain names, growth conditions and sources are summarized in the Supplementary Table S1.

### 4.8. Antibacterial Assays

The different bacterial and yeast strains were inoculated in 5 mL of the appropriate medium and incubated overnight at the appropriate temperatures and shaking at 200 rpm. Next, agar plates were overlaid with 5 mL soft agar (0.5%), seeded with 50 μL of the overnight cultures (~10^9^ CFU/mL). Cell lawns were supplemented with 5-μL spots of the different conotoxins and derivatives (concentration ~1 mM) and air-dried. Plates were incubated overnight and evaluated for the presence of zones of growth inhibition or halos. ND96 buffer was used as the negative control.

## 5. Conclusions

We purified five novel conotoxins from the venom glands of three Indian cone snail species that were largely unexplored up to now. The discovered sequences open new perspectives concerning the conopeptide classification and hint to new representatives of new classification groups since the amino acid sequences significantly differ from what is known in the literature. No targets could be attributed to the peptides, pointing to novel functionalities. Further experiments on other ion channels or receptors are required to reveal the physiological impact of these conopeptides.

## Figures and Tables

**Table 1:** Overview of the peptides discussed in this work.

Name	Amino Acid Sequence	Cysteine Arrangement	Cysteine Framework	Source
Lo6/7a	DQCSYCGIYCCPPKFCTSAGCRSP *	C–C–CC–C–C	VI/VII	*C. longurionis*
Lo6/7b	SCLSSGALCGIDSNCCNGCNVPRNQCY *	C–C–CC–C–C	VI/VII	*C. longurionis*
Asi3a	CCQWPCSHGCIPCCY *	CC–C–C–CC	III	*C. asiaticus*
Asi14a	SCGYPCSHCGIPGCYPG *	C–C–C–C	XIV	*C. asiaticus*
AusB	GAYFDGFDVPCVPRRDDC	C–C	N.A.	*C. australis*

* Indicates *C*-terminal amidation. N.A.: not available.

**Table 2:** List of yeasts, Gram-negative and Gram-positive bacteria used in antimicrobial screening of five different conopeptides.

	Gram-Negative Bacteria	Gram-Positive Bacteria	
1	*Aeromonas hydrophila* ATCC7966	1	*Bacillus megaterium* ATCC13632
2	*Agrobacterium tumefaciens* A208	2	*Bacillus subtilis* LMG 7135
3	*Azospirillum brasilense* Sp7	3	*Brevibacterium linens* ATCC9172
4	*Bordetella avium* 197N	4	*Corynebacterium glutamicum* DSM20300
5	*Brevundimonas diminuta* LMG 2088	5	*Lactobacillus plantarum* LMG-P21295
6	*Burkholderia cepacia* LMG 1222	6	*Lactobacillus rhamnosus* GG LMG 6400
7	*Burkholderia gladioli* LMG 2216	7	*Mycobacterium smegmatis* DSM43756
8	*Burkholderia vietnamensis* LMG 10927	8	*Rhodococcus erythropolis* N11
9	*Chromobacterium violaceum* CV026	9	*Staphylococcus aureus* ATCC6358
10	*Citrobacter freundii* ATCC8090	10	*Streptomyces lividans* TK24
11	*Enterobacter aerogenes* ATCC13048		
12	*Erwinia amylovora* CFBP1430	**Yeast**	
13	*Erwinia carotovora* LMG 2458	1	*Candida albicans* CAI4
14	*Proteus vulgaris* LMM2011	2	*Saccharomyces cerevisiae* W303-1A
15	*Pseudomonas aeruginosa* PA14		
16	*Pseudomonas entomophila* L48		
17	*Pseudomonas fluorescens* Pf0-1		
18	*Pseudomonas putida* KT2440		
19	*P. syringae* pv. *tabaci* LMG 5192		
20	*Rhizobium etli* CNPAF512		
21	*Salmonella enteritidis* ATCC13076		
22	*Serratia entomophila* DSM12358		
23	*Shigella flexneri* LMG 10472		
24	*Sphingomonas wittichii* RW1		
25	*Variovorax paradoxus* LMG 1797		
26	*Vibrio harveyi* BB120		
27	*Xanthomonas axonopodis* pv. *manihotis* LMG 784		
28	*Xanthomonas alfalfae* pv. *alfalfae* LMG 497		
29	*Yersinia enterocolitica* LMG 7899		

## Supplementary Materials

The following are available online at www.mdpi.com/1660-3397/14/11/199/s1, Table S1: Strain names, growth conditions (media and growth temperature) and source of the bacterial strains used in this work.
