# Supplementary material for: Novel Conopeptides of Largely Unexplored Indo Pacific Conus sp
Source: Mar Drugs. 2016 Oct 27;14(11):199. doi: 10.3390/md14110199 (PMC5128742; doi:10.3390/md14110199)
Supplement: Supplementary file 1 [file marinedrugs-14-00199-s001.docx]

**Supplementary Materials: Novel Conopeptides of Largely Unexplored Indo Pacific *Conus* sp.**

Eline K. M. Lebbe, Maarten G. K. Ghequire, Steve Peigneur, Bea G. Mille, Prabha Devi, Samuthirapandian Ravichandran, Etienne Waelkens, Lisette D'Souza, René De Mot and
Jan Tytgat

**Table S1.** Strain names, growth conditions (media and growth temperature) and source of the bacterial strains used in this work. LB, lysogeny broth; NB, nutrient broth; TSB, trypticase soy broth; YEP, yeast extract peptone; TY, tryptone yeast extract.

| **Strain Name** | | **Growth Conditions** | **Source** |
| --- | --- | --- | --- |
| Gram-Negative Bacteria | | | |
| *Aeromonas hydrophila* ATCC7966 | | LB, 30 °C | CMPG ^$^-collection |
| *Agrobacterium tumefaciens* A208 | | NB, 30 °C | CMPG-collection |
| *Azospirillum brasilense* Sp7 | | NB, 30 °C | Institut Pasteur |
| *Bordetella avium* 197N | | LB, 37 °C | CMPG-collection |
| *Brevundimonas diminuta* LMG 2088 | | TSB, 30 °C | BCCM * |
| *Burkholderia cepacia* LMG 1222 | | YEP, 30 °C | BCCM * |
| *Burkholderia gladioli* LMG 2216 | | YEP, 30 °C | BCCM * |
| *Burkholderia vietnamensis* LMG 10927 | | LB, 28 °C | BCCM * |
| *Chromobacterium violaceum* CV026 | | LB, 30 °C | CMPG-collection |
| *Citrobacter freundii* ATCC8090 | | NB, 37 °C | BCCM * |
| *Enterobacter aerogenes* ATCC13048 | | NB, 37 °C | BCCM * |
| *Erwinia amylovora* CFBP1430 | | LB, 30 °C | BCCM * |
| *Erwinia carotovora* LMG 2458 | | LB, 30 °C | BCCM * |
| *Proteus vulgaris* LMM2011 | | NB, 37 °C | CMPG-collection |
| *Pseudomonas aeruginosa* PA14 | | TSB, 30 °C | VUB ^£^ (P. Cornelis) |
| *Pseudomonas entomophila* L48 | | TSB, 30 °C | P. Cornelis |
| *Pseudomonas fluorescens* Pf0-1 | | TSB, 30 °C | G. Compeau [1] |
| *Pseudomonas putida* KT2440 | | TSB, 30 °C | C. Nieto [2] |
| *P. syringae pv. tabaci* LMG 5192 | | TSB, 30 °C | BCCM * |
| *Rhizobium etli* CNPAF512 | | TY, 30 °C | J. Sprent |
| *Salmonella enteritidis* ATCC13076 | | LB, 37 °C | BCCM * |
| *Serratia entomophila* DSM12358 | | LB, 30 °C | BCCM * |
| *Shigella flexneri* LMG 10472 | | NB, 37 °C | BCCM * |
| *Sphingomonas wittichii* RW1 | | LB, 28 °C | CMPG-collection |
| *Variovorax paradoxus* LMG 1797 | | LB, 28 °C | BCCM * |
| *Vibrio harveyi* BB120 | | LB, 37 °C | CMPG-collection |
| *X. axonopodis pv. manihotis* LMG 784 | | TSB, 30 °C | BCCM * |
| *X. alfalfa pv. alfalfae* LMF 497 | | TSB, 30 °C | BCCM * |
| *Yersinia enterocolitica* LMG 7899 | | LB, 30 °C | BCCM * |
| Gram-positive strains | | | |
| *Bacillus megaterium* ATCC13632 | NB, 30 °C | | CMPG-collection |
| *Bacillus subtilis* LMG 7135 | TY, 30 °C | | BCCM * |
| *Brevibacterium linens* ATCC9172 | LB, 30 °C | | CMPG-collection |
| *Corynebacterium glutamicum* DSM 20300 | TSB, 30 °C | | DSMZ ^§^ |
| *Lactobacillus plantarum* LMG-P21295 | MRS, 37 °C | | CMPG-collection |
| *Lactobacillus rhamnosus* GGLMG 6400 | MRS, 37 °C | | BCCM * |
| *Mycobacterium smegmatis* DSM 43756 | LB, 30 °C | | DSMZ ^§^ |
| *Rhodococcus erythropolis* N11 | LB, 30 °C | | BCCM * |
| *Staphylococcus aureus* ATCC6358 | TSB, 37 °C | | CMPG-collection |
| *Streptomyces lividans* TK24 | 10 g/L casitone, 5 g/L Yeast extract, 5 g/L glucose, 30 °C | | CMPG-collection |
| Yeast strains | | | |
| *Candida albicans* CAI4 | | YPD, 37 °C | CMPG-collection |
| *Saccharomyces cerevisiae* W303-1A | | YPD, 30 °C | CMPG-collection |

^$^ CMPG, Centre of Microbial and Plant Genetics; * BCCM, Belgian Coordinated Collections of Microorganisms [3]; ^£^ VUB, Vrije Universiteit Brussel. ^§^ DSMZ, Deutsche Sammlung von Mikroorganismen und Zellkulturen [4].

**References**

1. Compeau, G.; Al-Achi, B.J.; Platsouka, E.; Levy, S.B. Survival of rifampin-resistant mutants of *Pseudomonas fluorescens* and *Pseudomonas putida* in soil systems. *Appl. Environ. Microbiol.* **1988**, *54*, 2432–2438.
2. Nieto, C.; Fernandez-Tresguerres, E.; Sanchez, N.; Vicente, M.; Diaz, R., Cloning vectors, derived from a naturally occurring plasmid of *Pseudomonas savastanoi*, specifically tailored for genetic manipulations in Pseudomonas. *Gene* **1990**, *87*, 145–149.
3. BCCM. Belgian Coordinated Collections of Microorganisms. Available online: http:// belspo.be/bccm (accessed on 18 August 2016).
4. DSMZ. Deutsche Sammlung von Mikroorganismen und Zellkulturen. Available online: www.dsmz.de (accessed on 18 August 2016).
